# Leiomyosarcoma Invading the Vena Cava: A Case Report

**DOI:** 10.7759/cureus.22838

**Published:** 2022-03-04

**Authors:** Abdullah A Alsaghyir, Ghassan I Alhajress, Khaled Aldraihem, Abdullah Alhaidar, Ahmed I Nazer

**Affiliations:** 1 College of Medicine, King Saud Bin Abdulaziz University for Health Sciences, Riyadh, SAU; 2 College of Medicine, King Abdullah International Medical Research Center, Riyadh, SAU; 3 Division of Urology, Department of Surgery, Ministry of National Guard Health Affairs, Riyadh, SAU

**Keywords:** ivc tumor, atrium thrombus, colon leiomyosarcoma, intravenous leiomyosarcoma, kidney leiomyosarcoma

## Abstract

Leiomyosarcoma is an aggressive soft tissue tumor originating from smooth muscle cells typically of the uterus, gastrointestinal, or genitourinary system. The most common site of leiomyosarcoma of soft tissue is the retroperitoneum, accounting for 50% of all cases. The majority of patients are asymptomatic, which may be due to the large retroperitoneal cavity. However, when symptoms do occur, they are vague in nature. The most common growth pattern is an entirely extravascular mass. We are presenting an interesting case of a 65-year-old lady, who was referred to our hospital as a case of large left retroperitoneal mass with left renal vein thrombosis. She was biopsied and diagnosed with leiomyosarcoma with invasion into descending colon and the left renal vein, which led to renal vein thrombus. In the last few decades, there is a lack of studies about leiomyosarcoma invading the renal vein and Inferior Vena Cava (IVC). As far as we know, the leiomyosarcoma of a major blood vessel is extremely rare. Since leiomyosarcoma often has a late presentation with the advanced stage when detected, a high index of suspicion is needed to be detected early and avoid such a complication.

## Introduction

Leiomyosarcoma (LMS) is an aggressive soft tissue tumor originating from smooth muscle cells, typically of the uterus, gastrointestinal, or genitourinary system [1]. The most common site of LMS of soft tissue is the retroperitoneum, accounting for 50% of all cases. Retroperitoneal LMSs may grow large in size before they can be detected. LMS is usually discovered incidentally on imaging. The majority of patients are asymptomatic, which may be due to the large retroperitoneal cavity. However, when symptoms do occur, they are vague in nature [2]. Patients with retroperitoneal LMS can present with a wide range of symptoms including abdominal pain, fullness, or neurological symptoms [1]. The most common growth pattern is an entirely extravascular mass. Less commonly, LMS may demonstrate both extravascular and intravascular components. Rarely, retroperitoneal LMSs are completely intravascular, typically arising from the inferior vena cava (IVC) [2]. Due to the rarity of these tumors and the need for a multi-specialty treatment team, the treatment of LMS is best carried out in a specialized center with expertise in sarcoma care. Following that, a treatment plan is developed with the help of orthopedics, general surgeons, musculoskeletal radiologists, pathologists, medical oncologists, and radiation oncologists. In patients with localized single foci recurrences, whether local or metastatic, surgical resection should be strongly considered [3].

## Case presentation

**Figure 1:**
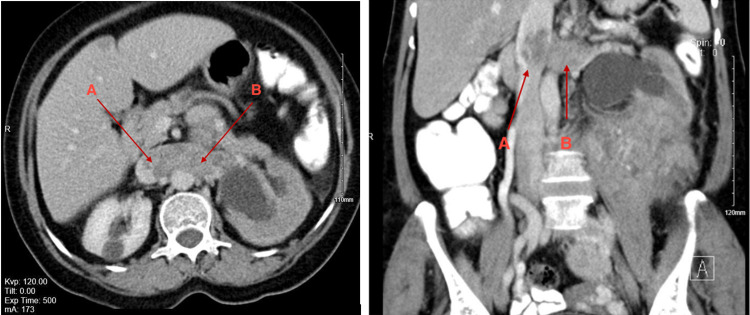
CT scan with contrast showed involvement of IVC and Left Renal Vein as pointed with the arrows. In both left-hand (axial view) and right-hand side (coronal) CT scan images- A: leiomyosarcoma invading inferior vena cava (IVC) B: leiomyosarcoma invading Left Renal Vein

We are presenting an interesting case of a 65-year-old lady, who was referred to our hospital as a case of large left retroperitoneal mass with left renal vein thrombosis. Her main complaint was on-off, vague abdominal pain. During the hospital course, she had two episodes of vomiting. She was fully investigated. Her lab results showed low hemoglobin (10.8 g/dl), low hematocrit (0.321%), high platelets (508 x 10^9^/L), low albumin (33 g/dl) and low sodium (130 mmol/L ). Positron Emission Tomography-Computed Tomography (PET-CT) was not done as it is not indicated in LMS. A bone scan was done and it showed no evidence of osseous metastasis. Chest CT showed no focal lung lesion. Detailed pre-operative abdomen and pelvis CT report showed approximately 20 x 14 cm large heterogenous mass of soft tissue attenuation displacing the retro-peritoneal structures located in the left inferior peri/para-nephric region with marked heterogeneous contrast enhancement and areas of necrosis. The mass demonstrated a clear line of cleavage between it and the lower pole of the left kidney in some images while in other images it could not be separated from the lower pole of the left kidney. The mass is multi-lobulated, extending from the left peri/para-nephric space down to the lower pelvis which lies in close contact with the left lateral wall of the uterus. The descending colon was displaced anteriorly and laterally by the mass, but no signs of obstruction were noted. Invasion of bowel lobes cannot be ruled out. The mass is also noted to extend into the left renal vein which is significantly distended and the intrahepatic portion of the IVC at the level of the renal vein (Figure 1). 

**Figure 2:**
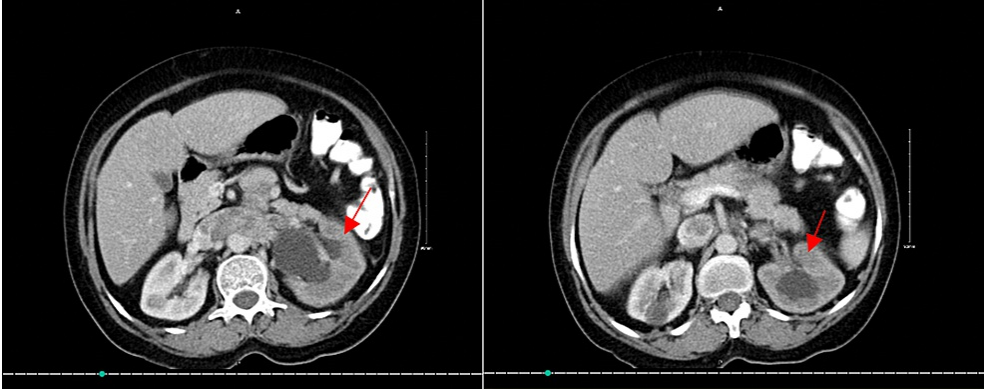
CT scan (axial view) showing Leiomyosarcoma as pointed by red arrows. Left-hand side image: Superior cut; Right-hand side image: Inferior cut

No areas or calcification were seen within the mass. Findings were associated with marked left hydronephrosis due to marked compression of the left ureter. She was biopsied and diagnosed with an LMS with invasion into descending colon and the left renal vein, which led to renal vein thrombus (Figure 2).

**Figure 3:**
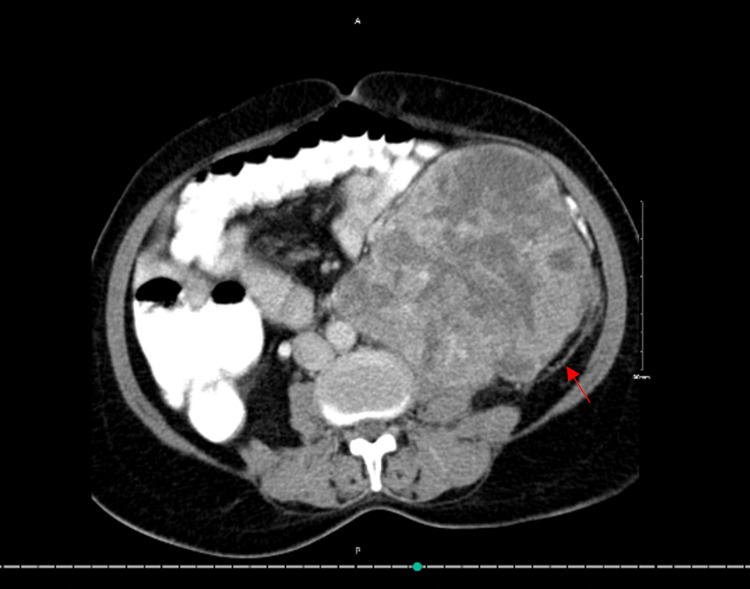
CT scan (axial view). The red arrow shows Leiomyosarcoma invading the nearby structures

**Figure 4:**
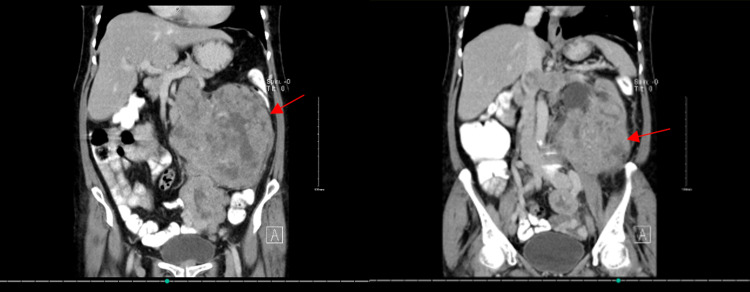
Coronal view showing Leiomyosarcoma compressing on nearby structures as pointed by red arrows. Left-hand side image: Posterior cut; Right-hand side image: Anterior cut

**Figure 5:**
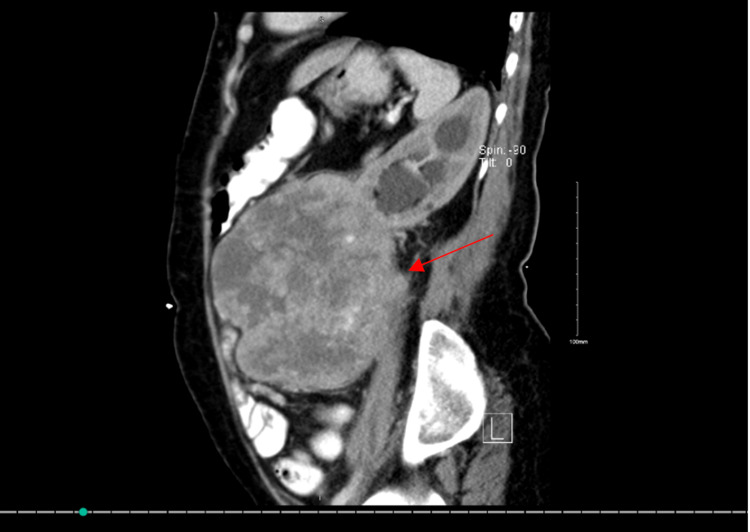
Lateral view showing Leiomyosarcoma compressing on nearby structures as pointed in the arrow.

The biopsy specimens were taken from the left renal vein, thrombus tumor, left kidney tumor, sigmoid, and spleen. The left renal vein showed a negative result for malignancy, while the thrombus tumor showed LMS. Furthermore, the specimen from the kidney, sigmoid colon, and spleen showed pleomorphic LMS and angioinvasion was identified. The case was discussed with the tumor board for the renal vein thrombus and extension in the IVC (Figures 3-5).

The resectability of the tumor was discussed thoroughly. Both the uro-oncologist and surgical oncologist saw a change in resecting the tumor completely and since surgical resection remains the standard of primary treatment of retroperitoneal sarcomas in general, the tumor board, which included medical and radiation oncologists, agreed to proceed with primary resection and possible adjuvant radiation. The patient was booked for a whole day of surgery. During the procedure, induction of anesthesia was smooth. A double-lumen endotracheal tube was used as the possibility of a thoracoabdominal approach was identified. The area was prepped and draped in a usual manner. The patient was put in a supine position where the upper thorax was on the break of the table. The upper thorax and the upper abdomen were opened wide, the tumor was obvious by a naked eye. A midline incision was done throughout the abdomen and the tumor was palpated. The surgical and vascular teams were involved from the beginning of the surgery. Due to its huge size and its invasion not only to the left mesocolon but also to the psoas muscle (Figure 3), the respectability of the tumor was questionable. At this point, the general surgery team suggested that it is LMS invading the renal vein, and the tumor was stuck (Figures 4,5), the decision was made that it is difficult to negotiate the vena cava before mobilizing the tumor because of its large size (Figure 5). So, the suggestion was to mobilize the tumor then control the vena cava. After that, dissection of the tumor was made from the psoas muscle, there was bleeding from the lumbars and the spleen. so, splenectomy was carried out and lumbars were ligated. 

As the dissection went on, both the tumor and the kidney were resected. Meanwhile, there was massive bleeding in between. The patient received around 10 units of blood. At this point, the tumor thrombus was suspected to be removed with the tumor or still in the vena cava, for which the vascular surgeon got the control above and below the renal vein. The renal vein seemed empty and the possibility of the tumor in the vena cava was still there. The inspection of the vena cava was through the renal vein but there was bleeding. Part of the tumor thrombus was excised. A suggestion that the thrombus might be avulsed to the heart was made. For that, a Fogarty catheter 6-french (Edwards Fogarty Catheters, California, USA) has been brought and passed up to the heart and then pulled out resulting in the unlikely possibility of the tumor being dislodged to the heart. At this point, the right ureter was injured, the decision and the repair were made by using the Boari flap. 

The tumor was completely resected and the decision for left colostomy was made. There was no bleeding, two drains were applied. During this period, the cardiology service was contacted to perform transthoracic echocardiography to role any tumor thrombus. And if there is any tumor thrombus the cardiac surgeon would be involved to remove the thrombus. Therefore, the patient was shifted in a stable condition from the Operating Room (OR) into the Intensive Care Unit (ICU) where the patient blood pressure dropped, and was suspected to have a massive pulmonary embolism. So, transthoracic echocardiography was done immediately on the table and there was a large thrombus on the right ventricle, but the patient was then crashing and having a pulmonary embolism. The resuscitation was continued for 40 minutes. Unfortunately, the patient could not be revived. The resuscitation was stopped and the patient was announced dead.

## Discussion

Leiomyosarcoma (LMS) is a rare and unpredictable smooth muscle malignancy. Soft tissue sarcomas account for 0.7 percent of all cancers, with LMS accounting for 5-10% [4]. LMSs can develop in soft tissue, skin, blood vessels, or bone, and the histology is the same regardless of location [4]. The retroperitoneum is the most common site of soft-tissue LMS, accounting for half of all cases [5]. Sarcoma metastasis is usually caused by hematogenous spread, so cutaneous metastasis is a rare finding [6]. When the retroperitoneum is involved, the most common symptoms are vague abdominal discomfort, an abdominal mass, and weight loss. Peripherally located masses may present as an enlarging mass, which is often painless and has few constitutional signs. Due to the deep inaccessible location and larger volume of the abdominal cavity, LMSs of the retroperitoneum tend to be significantly larger at presentation than those of the extremities [7].

Before the initiation of therapy, all patients with advanced retroperitoneal sarcomas should be evaluated and managed by a multidisciplinary team with expertise and experience in sarcomas. Retroperitoneal tumors should be categorized into resectable vs non-resectable or stage IV metastatic disease. For those that are deemed resectable there is no evidence favoring neoadjuvant over adjuvant chemotherapy or radiotherapy which should be individualized for each case.

In the last few decades, there is a lack of studies about LMS invading the renal vein and IVC. As far as we knew, LMS of a major blood vessel is extremely rare, with only a few hundred cases reported in the literature [8]. In 1871, the first case was published in German literature by Perl L where it was noticed in the sixth decade, with a feminine predominance [8]. Recently, around 505 cases of metastatic LMS have been reported in the literature [8]. In another case report conducted by Ivan et al., a 76-year-old woman underwent tumor resection and a microscopic examination revealed a moderate differentiated LMS originating from the wall of the renal vein [9]. Similarly, in a case report of a 46-year-old female presenting with bilateral leg swelling and abdominal distention, she was fully investigated and her CT, MRI, and color Doppler scan all revealed IVC, hepatic, and renal vein thrombosis. The histopathology of the thrombus indicated LMS with a confirmed involvement of the right atrium [8].

In our case report, we believe that all precautions were taken out since the diagnosis was made. A multidisciplinary team was involved from the beginning that included medical oncology and surgical oncology. However, death is always a possible outcome in a patient with a very large LMS especially when it involves the IVC.

## Conclusions

What is unique about our case is that LMS extended from the left renal vein into the inferior vena cava despite the tumor being on the left side. Consequently, the thrombus was dislodged in the right atrium. This finding suggests that since LMS is an aggressive soft tissue tumor and the majority of patients are asymptomatic along with its advanced stage when discovered. It is advisable to have a high index of suspicion to detect it early and avoid such complications.
